# Comparative finite-element analysis: a single computational modelling method can estimate the mechanical properties of porcine and human vertebrae

**DOI:** 10.1098/rsif.2014.0186

**Published:** 2014-06-06

**Authors:** K. Robson Brown, S. Tarsuslugil, V. N. Wijayathunga, R. K. Wilcox

**Affiliations:** 1 Imaging Laboratory, Department of Archaeology and Anthropology, University of Bristol, 43 Woodland Road, Bristol BS8 1UU, UK; 2 Institute of Medical and Biological Engineering, School of Mechanical Engineering, University of Leeds, Leeds LS2 9JT, UK

**Keywords:** finite-element analysis, vertebra, bone mechanics, porcine, human, comparative

## Abstract

Significant advances in the functional analysis of musculoskeletal systems require the development of modelling techniques with improved focus, accuracy and validity. This need is particularly visible in the fields, such as palaeontology, where unobservable parameters may lie at the heart of the most interesting research questions, and where models and simulations may provide some of the most innovative solutions. Here, we report on the development of a computational modelling method to generate estimates of the mechanical properties of vertebral bone across two living species, using elderly human and juvenile porcine specimens as cases with very different levels of bone volume fraction and mineralization. This study is presented in two parts; part I presents the computational model development and validation, and part II the virtual loading regime and results. This work paves the way for the future estimation of mechanical properties in fossil mammalian bone.

## 1. Introduction

Finite-element analysis (FEA) is a method of mathematical modelling widely used in engineering to underpin the design process by predicting the structural behaviour of components. In recent years, the method has also been used to estimate the mechanical response, in terms of stiffness and strength, of biological tissues such as bone to varied loading regimes [1–3], and many biologists have been quick to recognize the opportunities presented by FEA to explore the relationship between form and function in both living and extinct species [4–9]. There is a growing consensus in this field that finite-element (FE) models are most powerful and meaningful when they are validated against experimental data [5] and take account of the heterogeneous material properties of tissues [10–12]. Recent research in the field of vertebral biomechanics has demonstrated that FE models of spinal sections can achieve high levels of agreement in predicting mechanical characteristics when compared with corresponding specimens tested in the laboratory [13]. In these studies, the models have been generated from computed tomography (CT) images of the specimens which provide information on both the geometry and the material properties. This method is particularly attractive as a tool to evaluate bones from rare or extinct species where physical testing of the specimens is not possible [5,9,14]. Traditionally, the image greyscale is used to derive the bone density and hence mechanical properties. However, this approach is not possible with fossilized or dry bone since bone density calculations usually assume the trabecular space to be filled with marrow rather than air, soil or other matrix. Another approach is to use the bone volume fraction (bone volume over total volume, BV/TV), which can be derived from any state of bone provided the trabecular bone can be distinguished from the space on the images. This has been shown to provide models with good agreement for single species, but there is a need to prove the robustness over the full range of bone qualities if it is to be applied with confidence to evaluate palaeontological specimens.

We seek to develop a computational modelling method that can be shown to generate reliable estimates of the mechanical properties of bone across species, using elderly human and juvenile porcine specimens [15,16] as cases with very different levels of bone volume fraction and mineralization. This study is presented in two parts; part I presents the computational model development and validation, and part II the virtual loading regime and results.

## 2. Part I: computational model development and validation

### 2.1. Material and methods

Six porcine and four human cadaveric vertebrae which had been imaged and mechanically tested as part of other studies [13,17] were selected for the current work. The human cadaveric vertebrae were extracted from the spines of two donors aged 88 and 89 years, which were obtained from the local tissue bank following research ethics committee approval. The porcine vertebrae were harvested from the spines of two 6–8 month old pigs.

**Figure 1.**
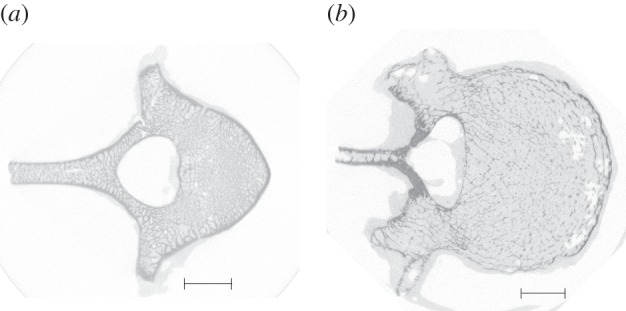
A μCT transverse image from (*a*) a porcine T12 specimen and (*b*) a human L4 specimen. The greyscale has been reversed for clarity such that darker regions show higher bone density. Scale bars, 10 mm.

The full details of the imaging and mechanical testing protocols have been reported previously [13,17]. Briefly, all of the specimens were frozen for storage and defrosted prior to testing. The vertebrae were stripped of soft tissue and the endplates embedded in polymethylmethacrylate (PMMA) cylindrical pots to provide flat parallel surfaces for testing. All of the specimens were imaged using micro-computed tomography (μCT) (Scanco μCT80, Scanco Medical, Brüttisellen, Switzerland) at a voxel size of 0.074 mm. Example μCT slices of each species are shown in figure 1. The same energy settings were used for all scans. The PMMA housings were left in place during imaging to allow their geometry to be accurately captured in the FE models. For each specimen, the vertebral body height and average cross-sectional area were determined from the μCT images.

Each specimen was then tested under axial compression in a materials testing machine (AGS-10kNG; Shimadzu Corp., Kyoto, Japan) at a loading rate of 1 mm min^−1^. The PMMA housings were fixed to steel end-caps and the load applied to the upper end-cap via a steel ball to allow rotation of the specimen during compression. Load and displacement data were saved during testing and the specimen stiffness was determined from the gradient of the load–displacement curve measured over a 0.6 mm interval.

### 2.2. Determination of image threshold values

It has been shown previously that good levels of accuracy can be achieved in specimen-specific FE models of vertebrae when the properties of each element within the model are derived from the bone volume fraction (BV/TV) in that region of the vertebral bone [18]. In order to determine the BV/TV values for each of the vertebrae, it was necessary to segment the images into bone and ‘background’ regions. Trabecular morphology varies from region to region within the vertebrae so in order to capture as much of the variation as possible the largest possible cuboidal region of interest (10 × 10 mm in the transverse plane and 20 mm axial depth) was extracted from the μCT images of the central region of each vertebral body. Histograms of the greyscale distribution were then calculated using a custom-written code.

**Figure 2.**
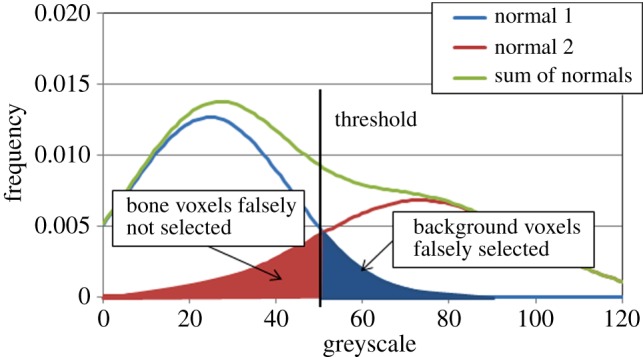
An illustration of the selection of the ‘optimum’ threshold. The histograms represent the normal distributions of greyscale in the background trabecular space and the bone. The selection of the ‘optimum’ threshold was undertaken such that the number of background voxels falsely classified as bone (light grey) was equal to the number of bone voxels falsely classified as background (dark grey). (Online version in colour.)

In all cases, there was overlap between the greyscale distribution of the trabeculae and that of the fluids within the trabecular space. It was also found that there was a marked difference in the greyscale distribution between the porcine and human specimens even though they were imaged using the same system with the same settings. This is likely to be due to the higher level of mineralization in the elderly human bone than in the juvenile pig bone. In order to derive an optimum species-specific threshold, a curve representing the sum of two normal distributions (with means *μ*_1_ and *μ*_2_ and variances 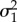 and 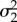) was fitted to the species average greyscale distribution by altering the values of the normal parameters (*μ*_1_, *μ*_2_, 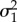 and 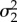) using a quasi-Newton iterative method (Solver, Microsoft Office Excel 2007, Microsoft Corp., Redmond, WA, USA). The two normal distributions were assumed to represent the trabeculae and the fluid in the trabecular space. In the case of the human specimens, there was a clear minimum between the two normal distributions, and this value was selected as the threshold. In the case of the porcine specimens, there was not a clear minimum between the two and the threshold was taken as the point at which the number of voxels that would be falsely classified as bone equalled the number falsely classified as trabecular space (figure 2).

In addition to the species-specific threshold values, a global threshold was also determined as the mean of the two species-specific values.

### 2.3. Model development

The derived species-specific threshold values were then applied to segment the images of all of the specimens of each species. In addition, a second set of segmented images of all of the specimens was generated by applying the global threshold to all of the scans from both species.

Each set of segmented images was then imported into an image-processing software tool (ScanIP, Simpleware Ltd, Exeter, UK) and downsampled to a resolution of 1 × 1 × 1 mm using an averaging method that allowed for partial volume effects. Thus, each downsampled voxel represented the average of the binary segmented voxels within it and therefore the greyscale value of the downsampled voxel represented the BV/TV value of that region of the underlying bone.

For each image set, the regions representing the vertebra and the PMMA housings were segmented on the downsampled images using a combination of threshold and floodfill operations along with closing (dilate–erode) procedures to fill small holes.

**Figure 3.**
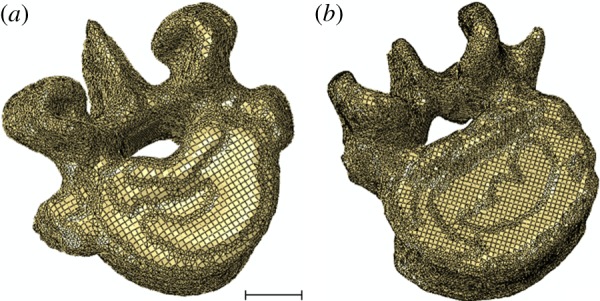
Typical FE models of (*a*) the porcine and (*b*) the human specimens. Scale bars, 10 mm. (Online version in colour.)

For all of the specimens, the regions were then imported into an FE meshing tool (ScanFE, Simpleware Ltd) and an FE model of the vertebra and two end-caps was generated. An element size of approximately 1 mm was used because this had previously been shown to be sufficient for vertebral stiffness evaluation in specimens of a similar size and under similar conditions [19]. A combination of hexahedral and tetrahedral linear elements was used to represent the vertebral geometry; in total, each model contained between 200 000 and 400 000 elements. Typical models of the two species showing the FE mesh are shown in figure 3.

The cement region was assigned an elastic modulus of 2.45 GPa [13]. Each element within the bone was assigned an elastic modulus based on the BV/TV value derived from the downsampled voxel greyscale. In previous studies of the vertebrae of single species, both linear and nonlinear relationships between the BV/TV value and the elastic modulus have been used [18,20]. Therefore, two relationships were investigated by generating model sets with the following formulae to relate the BV/TV value to the elastic modulus for each element:2.1
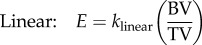
and2.2
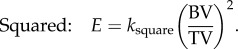


### 2.4. Model evaluation

All of the models were imported into an FE software package (ABAQUS CAE v. 6.9–1, Simulia Corp., Providence, RI, USA). Boundary conditions were applied to match the experimental test set-up. A single static point load was applied via a rigid plate to the upper cement endplate in the same position as the experiment. The interfaces between the bone and cement were assumed to be tied. The models were solved and the specimen stiffness determined. To normalize for size, the ‘apparent modulus’ was also determined by multiplying the stiffness by the vertebral height and dividing by the cross-sectional area. In total, four sets of 10 models were generated using either the global threshold or the species-specific threshold and either the linear or the square relationship between the BV/TV value and the elastic modulus for each element. All the models were processed and the predicted vertebral stiffness was determined.

For each of the four sets, comparisons were made between the model predictions and the experimental results by evaluating the mean absolute error in the stiffness and apparent modulus. In all cases, the optimum conversion factor *k* was determined by an iterative process until the mean error between the FE-predicted stiffness and the experimentally measured stiffness values across the whole set of specimens was minimized.

### 2.5. Results

**Figure 4.**
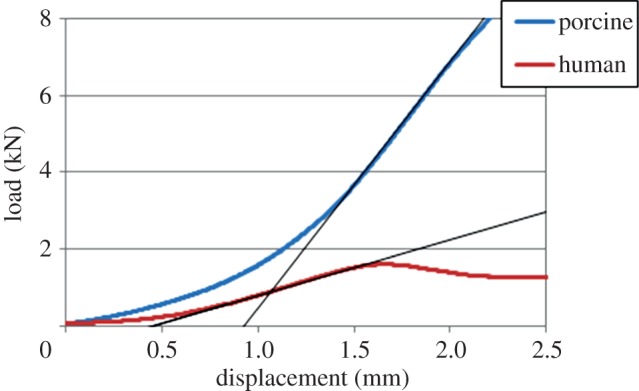
Typical load–displacement curves for a porcine (T12) and human (T7) specimen showing the linear regions from which the stiffness values were calculated. (Online version in colour.)

From the laboratory tests, the vertebral stiffness values were found to range from 0.9 to 1.5 kN mm^−1^ for the human specimens and from 5.3 to 6.4 kN mm^−1^ for the porcine specimens. Typical load–displacement graphs for the two species are shown in figure 4. There was a significant difference (Mann–Whitney non-parametric 2-tailed test at *p* ≤ 0.05) in both the vertebral stiffness and the vertebral apparent modulus between the human and porcine specimens.

From the computational results, it was found that, where the global threshold was applied across both species, the agreement in both the predicted stiffness and apparent modulus values with the corresponding experimental results was poor, with high levels of absolute error (table 1). This was found to be the case with the model sets generated with both linear and square relationships between the BV/TV value and the elastic modulus.

**Table 1.** Values of the average absolute error (=abs(FE stiffness – experimental stiffness)/(experimental stiffness)) between the FE-predicted stiffness and the experimental stiffness for each of the four sets of models.

method	same threshold for both species		optimized threshold for each species	
	linear	square	linear	square
optimized coefficient *k* (GPa)	0.32	1.03	0.33	0.78
average absolute percentage error (standard deviation in brackets)	61 (15)	44 (15)	21 (9)	26 (15)

Where a different threshold was used for each species, better agreement was achieved between the FE model predictions and the values obtained experimentally. The level of agreement was slightly higher and the error lower with the linear conversion factor than with the square conversion factor; in other words, the square relationship was not an improvement in terms of the comparison of the resulting models with the experimental data. In both cases, there was a significant difference (Mann–Whitney non-parametric 2-tailed test at *p* ≤ 0.05) in both the vertebral stiffness and the vertebral apparent modulus between the human and porcine models.

### 2.6. Conclusion to part I

From these results, the models of the porcine and human specimens derived using the species-specific threshold and the linear conversion factor were found to yield the lowest error compared with the experimental test cases. These 10 models were therefore used, employing this methodology, for the second part of the study.

## 3. Part II: loading regimes

### 3.1. Methods

**Figure 5.**
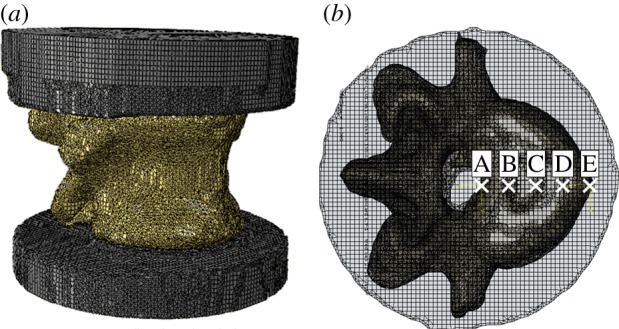
(*a*) An FE model after the inclusion of the cement end-caps and (*b*) a transverse view showing the location of the five loading positions. (Online version in colour.)

A series of virtual tests was then undertaken on the models generated with the species-specific threshold and the linear conversion factor. First, the height of the upper cement endcap was adjusted in all cases to be 40% of the vertebral body height to ensure the loading point was always the same relative distance from the vertebra. The load was then applied to five positions equally spaced between the anterior and the posterior extent of the vertebral body (figure 5). In each case, the model was solved and the vertebral stiffness determined as the load divided by the displacement at the point where the load was applied.

### 3.2. Results

**Figure 6.**
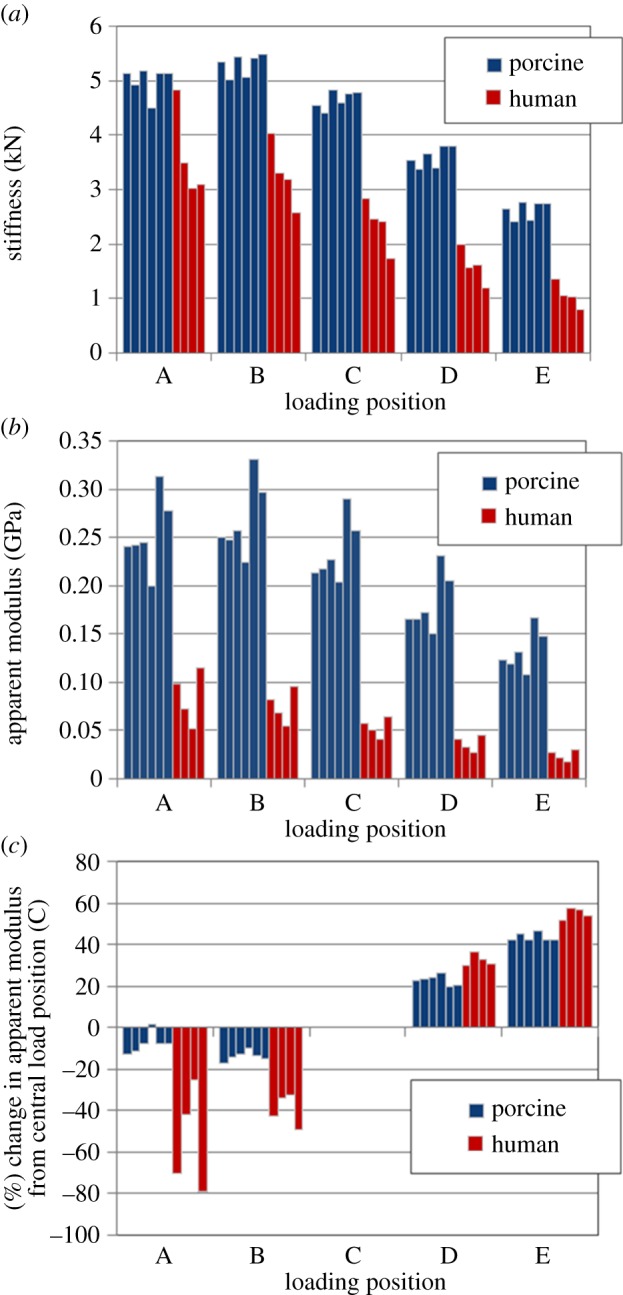
A comparison of the predicted values of (*a*) stiffness, (*b*) apparent modulus and (*c*) change in the derived modulus under different loading positions taken from the porcine and human vertebra FE models. (Online version in colour.)

The model-predicted stiffness and apparent modulus values for each vertebral model at each loading position are shown in figure 6. The load and displacement data for all FE models are deposited with Dryad (http://datadryad.org/). In all cases, the stiffness and apparent modulus increase as the loading position is moved from the anterior to the posterior of the vertebral body, owing to the increasing role of the neural arch. The change in stiffness was greater in the human specimens than in the porcine ones.

## 4. Discussion

The first objective of this project, to develop a consistent comparative method of computational modelling which could reveal the mechanical properties of vertebral bodies across species, was achieved through the development of a BV/TV method for deriving the elastic modulus which had previously been shown to generate models with good levels of accuracy (error approx. 10%) for single species [18]. The method was applied to two different mammalian species with different levels of bone tissue mineralization. The specimens of the two species were imaged at the same voxel size as was used in previous studies (0.074 mm), and the same resolution was used for all scans to maintain consistency of the BV/TV calculations. The thinnest trabeculae are approximately 0.15 mm in diameter, although most are thicker. The voxel size is sufficient to capture the whole trabecular structure and calculate BV/TV values to a reasonable level of accuracy. BV/TV calculations based on coarser voxel sizes have been shown to be correlated with those derived from a finer voxel size than that used in this study [21], so by maintaining the same voxel size for all scans in this study, any potential overestimation in the BV/TV value caused by the resolution was kept consistent and was therefore taken into account in the derivation of the elastic modulus conversion factor *k*. The levels of error of the resulting models were found to be higher than for a single species. This is not surprising because the method assumes that the elastic modulus is governed only by the BV/TV value, and differences in the underlying tissue modulus are not taken into account. It was apparent from the greyscale distributions of the porcine and human specimens that there was a clear difference between species, which is likely to be due to the higher level of mineralization, and therefore higher tissue modulus, in the mature human tissue than in the immature pig bone [22]. Despite this, when the conversion factor was optimized to reduce the error across both species, the levels of error were still sufficiently small to see a significant difference in both the predicted stiffness and apparent modulus between the two species. In this case, linear and square relationships were investigated because the optimum density–modulus relationships for FE models of trabecular bone have generally been found to lie in this range [23,24]. It was found that a linear relationship between the BV/TV value and the elastic modulus was slightly better than a square relationship. This is likely to be because the errors are dominated by the differences in tissue modulus and other factors such as the degree of anisotropy, so that any benefit of a potentially more accurate higher order relationship is not observed. It should be noted that, in all of the models, the cortex was represented using the same methodology as the trabecular bone, since the higher BV/TV in this region will produce elements with higher modulus values. This avoids the need to make additional assumptions about the cortex thickness or properties.

The analysis of the impact of loading position reveals species-specific features of the vertebral bodies. For both species the stiffness and apparent modulus increase as the loading position is moved from the anterior to the posterior of the vertebral body, owing to the increasing role of the neural arch. This is not surprising, given that in elderly humans the apophyseal joints of the neural arch have been shown to resist a significant proportion of compressive force applied to the thoracolumbar vertebra; on average 45%, but up to 96% in osteoarthritic specimens [25,26]. Interestingly, however, the change in stiffness with loading position was greater in the human specimens than in the porcine ones. This suggests that in pigs the posterior region may play a less significant role in resisting compressive vertebral loads than in humans, and it is likely that this reflects the quadrupedal posture of this species.

The computational modelling method presented here may be used to generate reliable estimates of the mechanical properties of bone across individuals and species, even where they present very different levels of bone fraction and mineralization. In practice, this means that in disciplines where model validation is difficult, such as palaeontology, this method could improve the level of confidence in model comparisons.
